# Construction and Evaluation of a Tumor Mutation Burden-Related Prognostic Signature for Thyroid Carcinoma

**DOI:** 10.1155/2021/1435827

**Published:** 2021-10-14

**Authors:** Haodong Lu, Qian Liu, Qing Chang, Jinghai Du, Chunying Zhang, Wang Chang, Xin Guo, Yaojie Hu, Shiguang Liu, Guoshuai Tang, Chunyou Chen

**Affiliations:** ^1^Department of Head and Neck Surgery, Tangshan Gongren Hospital, Tangshan, Hebei 063003, China; ^2^Shanghai Engineering Research Center of Pharmaceutical Translation, Shanghai 200231, China

## Abstract

Thyroid carcinoma is a type of prevalent cancer. Its prognostic evaluation depends on clinicopathological features. However, such conventional methods are deficient. Based on mRNA, single nucleotide variants (SNV), and clinical information of thyroid carcinoma from The Cancer Genome Atlas (TCGA) database, this study statistically analyzed mutational signature of patients with this disease. Missense mutation and SNV are the most common variant classification and variant type, respectively. Next, tumor mutation burden (TMB) of sample was calculated. Survival status of high/low TMB groups was analyzed, as well as the relationship between TMB and clinicopathological features. Results revealed that patients with high TMB had poor survival status, and TMB was related to several clinicopathological features. Through analysis on DEGs in high/low TMB groups, 381 DEGs were obtained. They were found to be mainly enriched in muscle tissue development through enrichment analysis. Then, through Cox regression analysis, a 5-gene prognostic signature was established, which was then evaluated through survival curves and receiver operation characteristic (ROC) curves. The result showed that the signature was able to effectively predict patient's prognosis and to serve as an independent prognostic risk factor. Finally, through Gene Set Enrichment Analysis (GSEA) on high/low-risk groups, DEGs were found to be mainly enriched in signaling pathways related to DNA repair. Overall, based on the TCGA-THCA dataset, we constructed a 5-gene prognostic signature through a trail of bioinformatics analysis.

## 1. Introduction

With the universalness of imaging for diagnosis and monitoring, the thyroid carcinoma (THCA) cases have been increasing throughout the world [1]. Although most patients with thyroid carcinoma respond to therapies like surgical intervention and iodine-131, 10% of them succumb to poor prognosis and malignant progression [2]. It can be perceived that diagnostic and therapeutic strategies for malignant thyroid carcinoma remain to be improved. Hence, accurate diagnosis and assessment of tumor malignancy, as well as personalized therapeutic options, may aid in improving the survival status of patients with this disease. Tumor mutation burden (TMB) is being defined as the number of somatic mutations per megabase of genome, which varies across malignancies [3]. Currently, TMB is broadly recognized to be associated with DNA damage repair (DDR). For instance, in 2018, Nassar et al. [4] found that DDR-related genes are correlated with TMB in a cohort study for gene mutational features in urothelial carcinoma. That is, the severer the loss of DDR-related gene function, the higher the corresponding TMB is in patients. Likewise, Parikh et al. [5] revealed that loss of DDR function is positively related to TMB. It is well known that loss of DDR function results in malignant progression of tumors [6], thus, TMB can partially reflect tumor malignancy. Besides, several studies analyzed the correlation between TMB and prognosis of patients through bioinformatics methods. For example, Xie et al. [7] concluded that compared with low-TMB patients, the prognosis is more unfavorable in high-TMB patients. Overall, there is a correlation between TMB level and prognosis of patients with thyroid carcinoma.

Screening gene signatures based on public databases is an effective strategy to search for prognostic biomarkers. Nowadays, researchers have searched for possible prognostic biomarkers based on varying features of multiple cancer types and have constructed various prognostic models, which provide relevant evidence for the prognosis of patients with related cancers. An example is that Liu et al. [8] generated a prognostic model of autophagy-related genes in nonsmall cell lung cancer. Guo and He [9] analyzed gene expression in the SLC30A family and manifested a likely prognostic marker for gastric cancer. Sui et al. [10] generated an immunocyte-related prognosis model that can predict the prognosis of patients effectively and efficacy of chemotherapy by analyzing immunocyte infiltration level in breast cancer. However, TMB-related prognostic model for thyroid carcinoma has not been fully studied yet.

Here, based on the dataset from The Cancer Genome Atlas (TCGA) database, we first perform statistical analysis on the mutational signature of thyroid carcinoma. Then, we analyzed relationships among TMB, survival time, and several clinicopathological features of patients. Afterward, we conducted Gene Ontology (GO) and Kyoto Encyclopedia of Genes and Genomes (KEGG) enrichment analyses on differentially expressed genes (DEGs) in high/low TMB groups. Based on these DEGs, we generated a 5-gene prognostic signature for thyroid carcinoma. In conclusion, the establishment of the prognostic signature may present an opportunity for its clinical application as a prognosticator of patients with this disease.

## 2. Methods

### 2.1. Data Acquisition and Mutational Signature Analysis

TCGA-THCA dataset was downloaded from TCGA (https://portal.gdc.cancer.gov/) database. The dataset included mRNA expression data (normal: 58; tumor: 510), single nucleotide variants (SNV) data (VarScan2 Annotation, tumor: 487), and common clinical profiles. The R package “maftools” [11] was utilized to conduct mutational signature analysis on samples of thyroid carcinoma patients in the TCGA-THCA dataset. This statistical analysis included variant classification, SNV class, variants frequency, and mutated gene of samples.

### 2.2. Correlation Analysis of TMB in Thyroid Carcinoma and Clinicopathological Features

The number of somatic mutations (nonsynonymous mutations) per megabase in the coding region of tumor genome in samples was defined as TMB value. Samples were stratified into high- and low-TMB groups per the upper quartile of TMB value. “Survival” package [12] was employed to plot survival curves of high/low TMB groups. Correlation between various clinicopathological features and TMB was analyzed and the variability was detected by Wilcoxon.

### 2.3. DEGs and Enrichment Analyses

The R package “edgeR” [13] was used to conduct differential expression analysis (∣logFC | >1, *p*adj < 0.05) on mRNA expression data in high/low TMB groups. TMB-related DEGs were obtained. The R package “clusterprofiler” [14] was employed to carry out GO and KEGG enrichment analyses on DEGs. Terms and signaling pathways subjected to *p* value < 0.05 and *q* value < 0.05 were selected as significantly enrichment results.

### 2.4. Establishment of the TMB-Related Prognostic Signature

Among TCGA-THCA samples, those with survival time greater than 0 were chosen and randomly grouped into training set (*n* = 350) and test set (*n* = 151). Univariate Cox regression analysis was carried out in the training set by using R package “survival,” and genes related to survival were obtained (*p* < 0.05). These genes were then subjected to LASSO regression analysis. Cross-validation was conducted to select the optimal penalty parameter (lambda) to rule out genes with relatively high correlation. Afterward, the “survival” package was applied for multivariate Cox regression analysis on the selected genes by LASSO analysis. The prognostic signature was constructed, by which risk score of each patient was computed following the formula:

(1) $$\begin{array}{c} \text{Risk score}=\overset{n}{\underset{j=1}{\sum }}\text{C}\text{oⅇ}{\text{f}}_{j}*\text{Gene expressio}{\text{n}}_{j}. \end{array}$$

*n* represents the number of prognosis-related genes. Coⅇf_*j*_ represents weighted correlation coefficients of each gene. Gene expression_*j*_ represents the expression of prognosis-related genes.

### 2.5. Prognostic Signature Evaluation and Gene Set Enrichment Analysis (GSEA)

In the training set, samples were classified into high- and low-risk groups with the median risk score of patients as the cut-off value. The R package “survival” was utilized to plot survival curves of high- and low-risk groups in the training set and the test set, respectively. Then, survival status was observed. The R package “timeROC” [15] was employed to draw receiver operation characteristic (ROC) curves in two sets, respectively, thereby evaluating the performance of the prognostic signature. Afterward, risk score was considered as a feature, combined with common clinicopathological features (sex, age, T staging, N staging, and tumor stage), univariate and multivariate Cox regression analyses were done. Next, R package “rms” [16], combined with clinical information of patients and risk score, was utilized to draw a nomogram of 3-year and 5-year survival. Finally, the R package “foreign” (https://cran.r-project.org/web/packages/foreign/index.html) was used to plot calibration curves to validate performance of the nomogram. Besides, to investigate main signaling pathways that variated across samples in high- and low-risk groups, GSEA software was downloaded from website (http://www.gsea-msigdb.org/gsea/index.jsp) for analysis of the expression profiles of patients in high- and low-risk groups [17].

## 3. Results

### 3.1. Statistical Analysis on Mutational Signature of Thyroid Carcinoma

**Figure 1:**
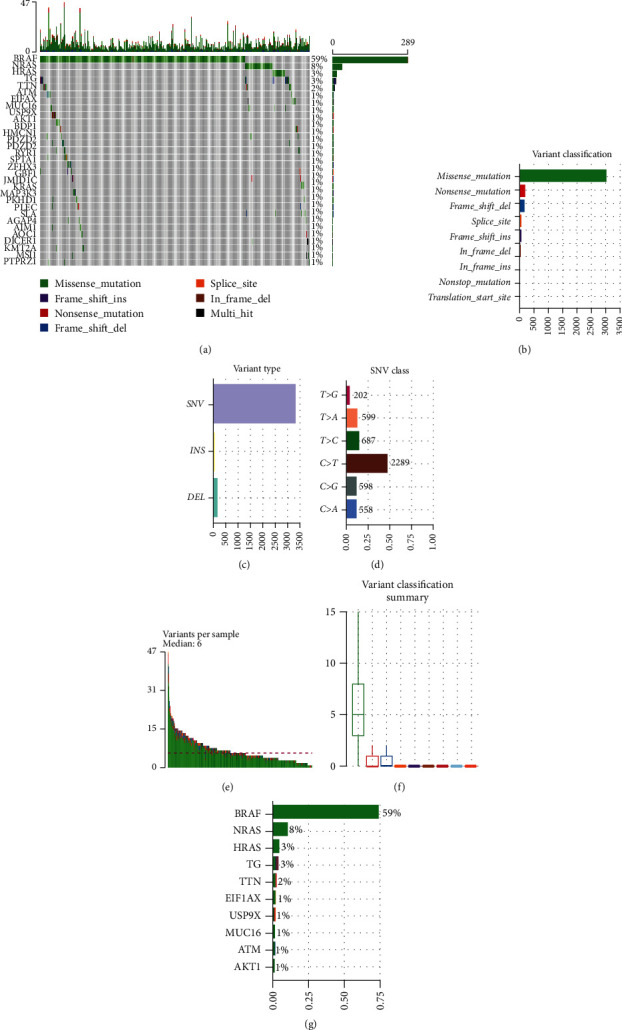
Statistical analysis on mutational signature of thyroid carcinoma. (a) Waterfall plot of the top 30 mutated genes in each sample. The abscissa represents different samples, and the ordinate represents the top 30 genes. The filled color represents that the gene mutated in the sample, and different colors represent different variant classifications. (b)–(d) According to different classification methods, variant classification, variant types, and SNV class were analyzed. SNV stands for single nucleotide variants. INS stands for insertion. DEL stands for deletion. (e) Histogram indicates the total number of variants per sample of thyroid carcinoma. (f) Box plot shows the distribution of variant classification in cancer samples. (g) Top 10 mutated genes in thyroid carcinoma samples.

To probe the mutational signature of thyroid carcinoma, R package “maftools” was utilized to visualize mutation information of 487 samples from TCGA-THCA. Waterfall plot revealed top 30 mutated genes in each sample (Figure 1(a)). Missense mutation was the most common (Figures 1(b) and 1(f)) in variant classification; SNV was the most common (Figure 1(c)) in variant type; the mutation from C to T was the most common (Figure 1(d)) in SNV class. Besides, the median of variants per sample was 6 (Figure 1(e)). The top 10 mutated genes were *BRAF* (59%), *NRAS* (8%), *HRAS* (3%), *TG* (3%), *TTN* (2%), *E1F1AX* (1%), *USP9X* (1%), *MUC16* (1%), *ATM* (1%), and *AKT1* (1%) (Figure 1(g)). To sum up, we believed that during the progression of thyroid carcinoma, there was a specific trend in the occurrence of mutations.

### 3.2. Correlation Analysis of TMB and Clinicopathological Features

**Figure 2:**
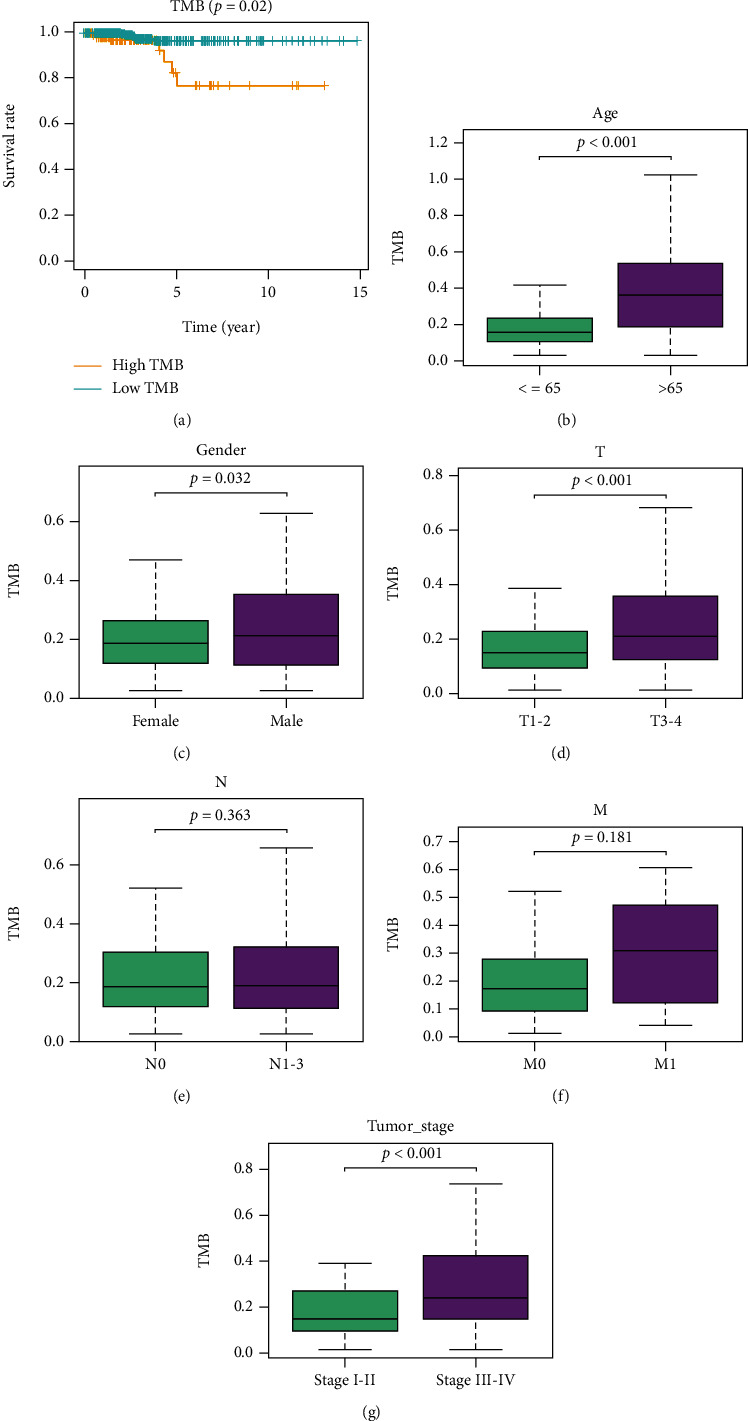
Correlation analysis of TMB and clinicopathological features. (a) Survival curves of patients in high/low TMB groups. (b)–(g) Correlation of TMB and clinicopathological features (age, sex, *T* staging, *N* staging, *M* staging, and stage).

The number of somatic mutations per megabase in the coding region of tumor genome is termed as TMB. TMB value of each sample was calculated to investigate the relationship between TMB and clinicopathological features of patients. Then, thyroid carcinoma samples were classified into high- and low-TMB groups per the upper quartile. Subsequently, survival curves were plotted and survival status in high/low TMB groups was observed. As illustrated in Figure 2(a), compared with high TMB group, survival of patients in low TMB group was more favorable. Furthermore, the correlation of TMB and various clinicopathological features was analyzed. The results presented that TMB was conspicuously upregulated in patients older than 65 (Figure 2(b)). TMB values in male patients were notably higher than those in female patients (Figure 2(c)). TMB values in patients in *T*3 − 4 were markedly higher than those of patients in *T*1 − 2 (Figure 2(d)). TMB values in patients in N1-3 were markedly higher than those of patients in *N*0 (Figure 2(e)). TMB values in patients in *M*1 were markedly higher than those of patients in *M*0 (Figure 2(f)). TMB values in patients in stage III-IV were remarkably higher than those of patients in stage I-II (Figure 2(g)).

### 3.3. DEGs and Enrichment Analyses

**Figure 3:**
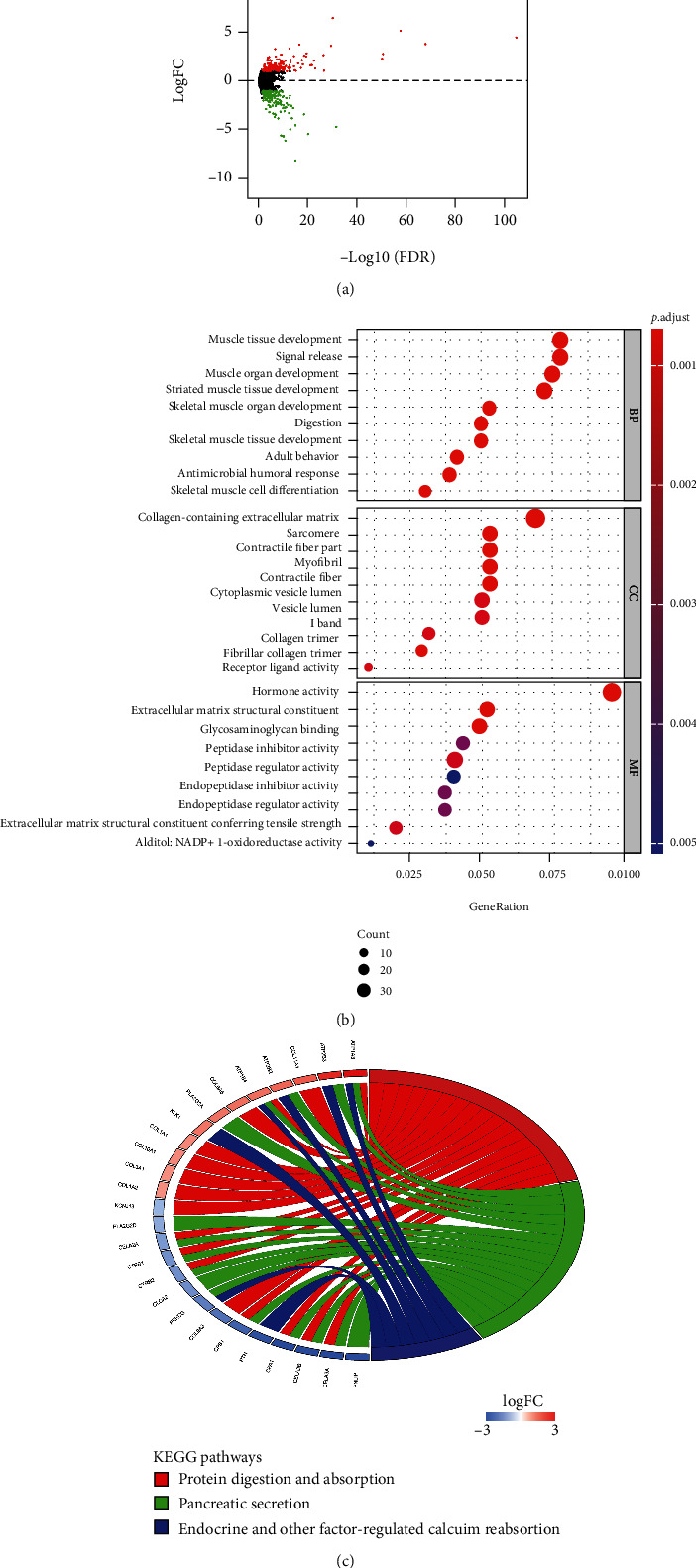
DEGs and enrichment analyses. (a) Volcano map of DEGs in high- and low-TMB groups (red dots represent notably upregulated genes; green dots represent notably downregulated genes). (b) Bubble chart of DEGs in GO enrichment analysis. The color of nodes with a small *p* value tends to be red; as the number of genes in the enrichment term increases, the size of nodes also increases. (c) Results of KEGG enrichment analysis of DEGs. The left half circle presents the genes enriched in each signaling pathway. Red represents upregulation, and blue represents downregulation. The color of gene with a larger |logFC| tends to be darker. The right half circle represents the main enriched signaling pathways, and each signal pathway is represented by a different color.

Samples with mRNA expression data were classified into high-TMB group (130) and low-TMB group (352) per the upper quartile of TMB. DEGs were analyzed. A total of 381 DEGs were obtained including 189 notably upregulated genes and 192 notably downregulated genes (Figure 3(a)) (Supplementary Table 1). Afterward, GO and KEGG enrichment analyses were performed on 381 DEGs. GO enrichment analysis revealed that these DEGs were mainly enriched in terms such as muscle tissue development, collagen-containing extracellular matrix, and receptor-ligand activity (Figure 3(b)). KEGG enrichment analysis manifested that these DEGs were mainly enriched in signaling pathways such as protein digestion and absorption, pancreatic secretion, endocrine and other factor-regulated calcium reabsorption (Figure 3(c)). Thus, we speculated that these DEGs were mainly involved in biological functions and signaling pathways related to muscle tissue development.

### 3.4. Construction and Assessment of the Prognostic Signature

**Figure 4:**
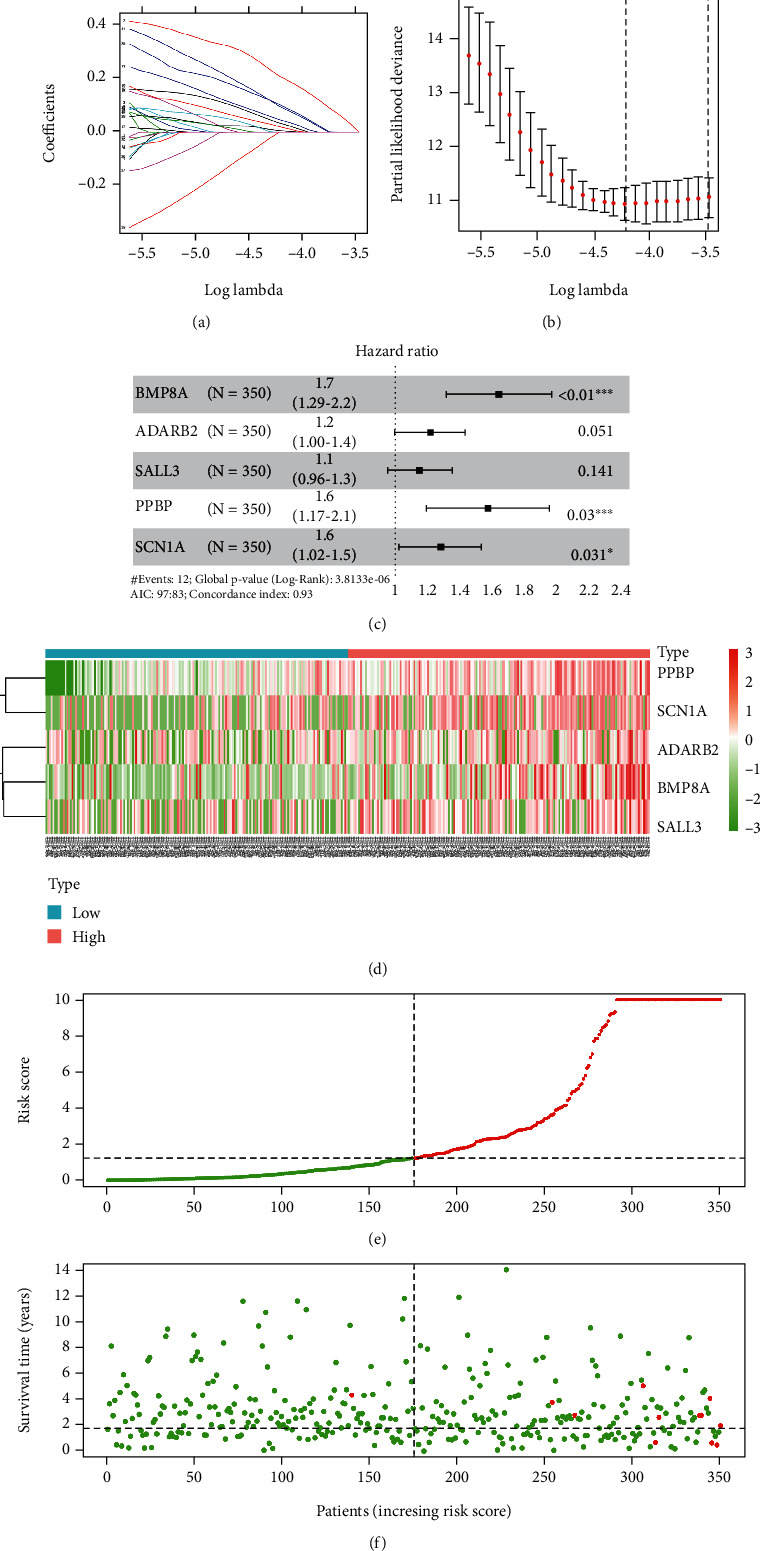
Prognostic signature construction. (a) The coefficients of 33 survival-related genes in LASSO analysis changes as the penalty parameter lambda changes. (b) In the LASSO regression model, when the optimal lambda is adjusted, the number of genes with a nonzero coefficient is seven. (c) Multivariate Cox regression analysis for generation of the 5-gene prognostic signature. (d) Heat map of the expression of 5 feature genes in high- and low-risk groups. (e) The distribution chart of risk scores of patients with thyroid carcinoma (red represents high-risk group, and green represents low-risk group). (f) The distribution chart of survival of patients with different risk scores (red represents the dead patients, and green represents the surviving patients).

Based on the above DEGs, to construct TMB-related prognostic signature for thyroid carcinoma, tumor samples from the TCGA-THCA dataset were divided into the training set (*n* = 350) and the test set (*n* = 151). To initially screen survival-related genes, 33 survival-related genes were obtained through univariate Cox regression analysis in the training set (Supplementary Table 2). Next, LASSO Cox regression analysis was conducted to further screen out 7 genes from 33 genes. Thus, genes that were too closely related to each other were excluded (Figures 4(a) and 4(b)). Finally, based on the above 7 genes, a 5-gene prognosis signature for thyroid carcinoma was established via multivariate Cox regression analysis (risk score = 0.5117∗BMP8A + 0.1719∗ADARB2 + 0.1205∗SALL3 + 0.4577∗PPBP + 0.2235∗SCN1A) (Figure 4(c)). Afterward, based on the prognostic signature, risk score of each sample in the training set was calculated. Patients with thyroid carcinoma were divided into high- and low-risk groups with the medium risk score of the training set as the cut-off value. Survival of patients and expression of 5 feature genes were analyzed (Figures 4(d)–4(f)).

**Figure 5:**
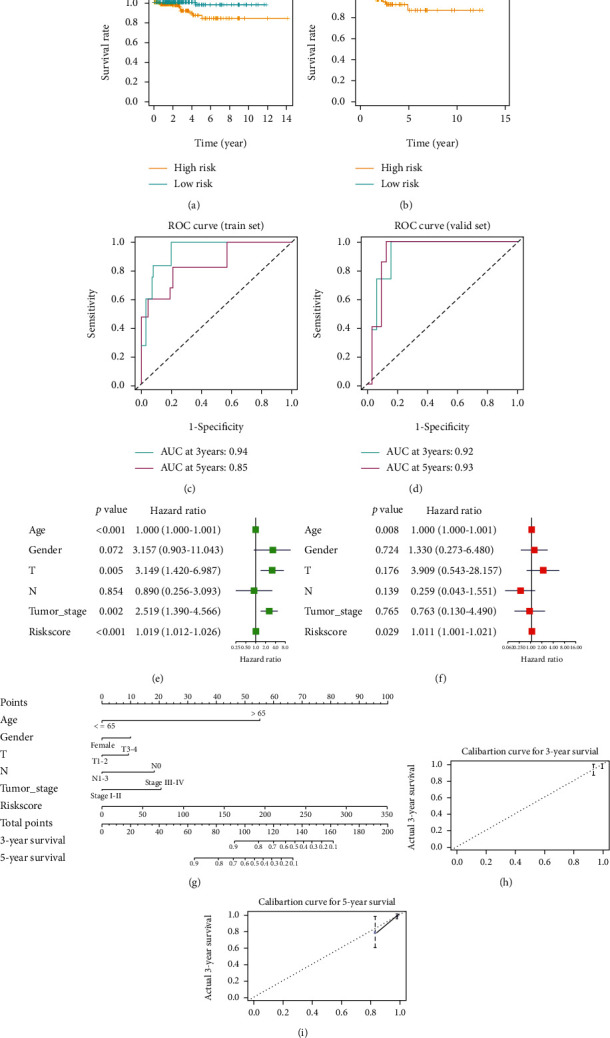
Evaluation of efficacy and independence of the prognostic signature. (a) Survival analysis on the high- and low-risk groups in the training set. (b) Survival analysis on the high- and low-risk groups in the test set. (c) In the training set, ROC curves were plotted based on the prognostic signature. (d) In the test set, ROC curves were plotted based on the prognostic signature. (e) Combined with common clinicopathological features, univariate Cox regression analysis was conducted on risk scores. (f) Combined with common clinicopathological features, multivariate Cox regression analysis was conducted on risk scores. (g) Combined with common clinicopathological features and risk score, a nomogram was plotted to predict 3-year and 5-year survival of patients. (h) and (i) The performance of the nomogram was validated through calibration curves.

To assess the performance of the prognostic signature, as well as its independence as a prognosticator, survival curves were plotted in the training set and the test set. Subsequently, survival analysis on patients in high- and low-risk groups was conducted. The results disclosed that survival of patients in the low-risk group was conspicuously better than those in the high-risk group (Figures 5(a) and 5(b)). Then, ROC curves were drawn in the training set and the test set to assess the performance of the prognostic signature. As illustrated in Figures 5(c) and 5(d), the 3-year and 5-year area under the curve (AUC) of the training set were 0.94 and 0.85, respectively. The 3-year and 5-year AUC of the test set were 0.92 and 0.93, respectively. A good performance of the prognostic signature was presented. Afterwards, combined with common clinicopathological features, univariate and multivariate Cox regression analyses were carried out on risk scores of patients. The results presented that in univariate Cox regression analysis, T staging, tumor stage, and risk score were markedly correlated with patient's prognosis. While in multivariate Cox regression analysis, only risk score remarkably affected patient outcomes (Figures 5(e) and 5(f)). Hence, according to results of univariate and multivariate Cox regression analyses, risk score could be an independent prognostic risk indicator. Finally, a nomogram was plotted to predict 3-year and 5-year survival of patients with thyroid carcinoma by combining various clinicopathological features and risk scores (Figure 5(g)). Calibration curves were drawn to evaluate the performance of the nomogram (Figures 5(h) and 5(i)). It could be seen that 3-year and 5-year survival of patients could be effectively predicted by the nomogram.

### 3.5. GSEA

**Figure 6:**
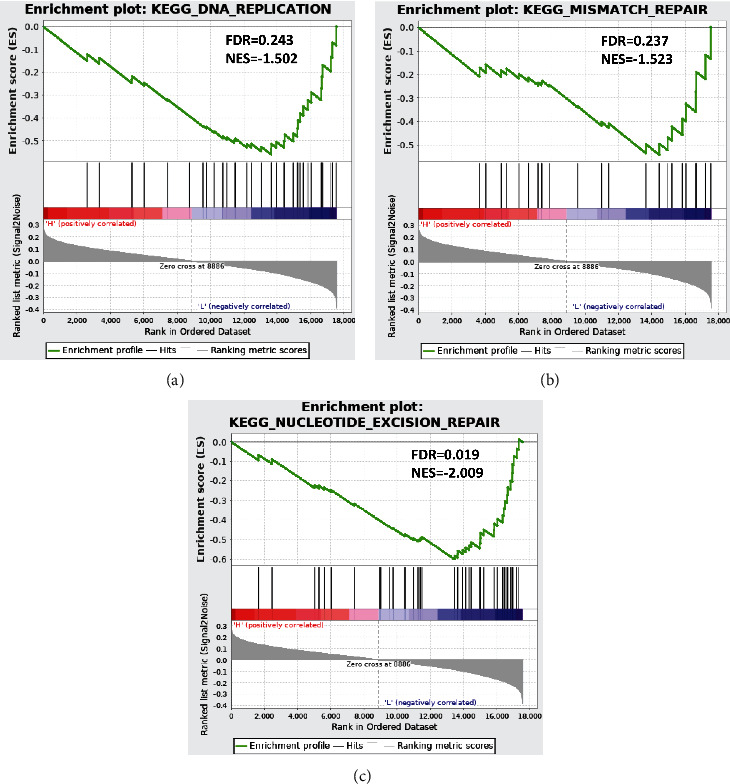
GSEA enrichment analysis in high- and low-risk groups. Enrichment of high- and low-risk groups in DNA replication pathway (a), mismatch repair pathway (b), and nucleotide repair pathway (c).

To inquiry about signaling pathways varied in patients in high- and low-risk groups, GSEA was performed on high- and low-risk groups. As presented in Figures 6(a)–6(c), significant differences showed in activation of signaling pathways including nucleotide excision repair, mismatch repair, and DNA replication in high- and low-risk groups. In conclusion, differentially activated signaling pathways may be responsible for prognosis difference between high- and low-risk groups.

## 4. Discussion

Based on the TCGA-THCA dataset, this study carried out statistical analysis on mutation information of thyroid carcinoma. *BRAF* gene mutation was the most common one. *BRAF*, as a member of serine/threonine protein kinase family, participates in MAPK/ERK signaling pathway in cells, and its mutation is related to thyroid canceration [18]. Numerous investigations mentioned high mutation rate of *BRAF* gene in various thyroid carcinomas [19–22], wherein T1799A nucleotide transversion is the most common oncogenic mutation of *BRAF* gene. This mutation leads to conversion of the 600th valine of *BRAF* protein to glutamate, which enhances the activity of serine/threonine protein kinase of *BRAF* protein. Overall, both mutational signature analysis in this study and earlier studies exhibited that mistranslation mutations of *BRAF* gene in thyroid carcinoma mutations play a pivotal role in thyroid carcinogenesis.

TMB varies in different solid tumors. In this study, TMB of patients with thyroid carcinoma was in the range of 0.02~1.63 (Supplementary Table 1). Nevertheless, literature reported that 50% of patients with lung squamous cell carcinoma and 71% of patients with melanoma have a TMB greater than 10. Investigators speculated that high TMB of the abovementioned solid tumor patients mainly results from long-term exposure to chronic mutagenic exposures (smoking or ultraviolet radiation) and long-term superimposed mutations [3]. Notwithstanding the limited floating range of TMB in thyroid carcinoma, our analysis illustrated a significant difference in survival time of patients in high and low TMB groups. That is, the survival of patients in the high TMB group is dismal (Figure 2(a)). As such, in 2020, Xie et al. [7] supported the view through a series of bioinformatics analyses that patients with papillary thyroid carcinoma with high TMB have unfavorable outcomes. Hence, combined our results with published literature, we could speculate that the prognosis of thyroid carcinoma patients with high TMB is poor.

According to American Joint Committee on Cancer staging manual eighth edition, in the TNM staging of thyroid carcinoma, T3 of thyroid carcinoma is defined as tumor > 4 cm limited to the thyroid, or gross extrathyroidal extension invading only strap muscles [23]. Meanwhile, a study [24] pointed out that the probability of local recurrence can be predicted according to strap muscle invasion of thyroid carcinoma. Hence, strap muscle invasion may relate to malignancy of thyroid carcinoma. In this investigation, DEGs were obtained by differential expression analysis on patients with high and low TMB. These genes were subjected to GO and KEGG enrichment analyses, and the results revealed that they were mainly enriched in biological functions related to muscle tissue development. Based on results of our analysis and published literature, we speculated that malignant progression of thyroid carcinoma affected the development of peri-thyroidal muscles.

GSEA manifested that patients in high- and low-risk groups mainly differed in DNA repair. Most studies exhibited the same trend with our results. In 2020, Ricciuti et al. [25] proposed that compared with the DDR-negative group, patients in the DDR-positive group show higher TMB. Besides, Chalmers et al. [26] indicated that mutations in mismatch repair signaling pathways-related genes often occur in high TMB cancers through an analysis of genomes from 100,000 patients with different cancers. These findings exhibited that dismal prognosis of patients in the high-risk group may be related to DDR-related gene mutations, which are related to high TMB.

All in all, based on thyroid carcinoma data set from public databases, this study performed statistical analysis on mutational signature of the disease. Samples were classified into high and low TMB groups based on the mutation data. Then, survival of patients in high and low TMB groups was compared, as well as common clinicopathological features. Meanwhile, DEGs of these two groups were subjected to GO and KEGG enrichment analyses. Finally, based on the obtained DEGs, a 5-gene prognostic signature was established by Cox regression analysis and LASSO analysis. The performance and independence of the prognostic signature were assessed. High- and low-risk scores calculated by the signature were subjected to GSEA. Although complete bioinformatics analyses were performed on thyroid carcinoma dataset and valuable results were obtained, the study was insufficient. For instance, we failed to explore the role of the 5 feature genes (BMP8A, ADARB2, SALL3, PPBP, and SCN1A) in onset and progression of thyroid carcinoma by molecular experiments, cell experiments, or animal experiments. Hence, further experiments need to be designed to probe the impact of these genes on malignant progression of thyroid carcinoma.

## Data Availability

All data generated or analyzed during this study are included in this published article.

## Supplementary Materials

Supplementary 1 Supplementary Table 1: DEGs in high- and low-TMB groups.

Supplementary 2 Supplementary Table 2: univariate Cox regression analysis on TMB-related DEGs.
