# Supplementary material for: Construction and Evaluation of a Tumor Mutation Burden-Related Prognostic Signature for Thyroid Carcinoma
Source: Comput Math Methods Med. 2021 Oct 14;2021:1435827. doi: 10.1155/2021/1435827 (PMC8538398; doi:10.1155/2021/1435827)
Supplement: Supplementary 1 — Supplementary Table 1: DEGs in high- and low-TMB groups. [file 1435827.f1.pdf]

GeneID	logFC	logCPM	PValue	FDR
IGF2	4.432853	7.120853	5.58E-109	9.79E-105
ATP1A3	3.754047	1.234196	4.42E-72	3.88E-68
CLEC4M	5.100429	-2.30878	8.91E-62	5.22E-58
ALK	2.736026	3.279239	1.29E-54	5.66E-51
ASAH2	2.258575	0.769492	3.67E-54	1.29E-50
MATN1	-4.63704	2.505861	1.67E-35	4.90E-32
MYH2	6.391607	1.509254	4.72E-34	1.18E-30
TERT	3.561382	-2.9179	2.63E-33	5.77E-30
THADA	1.049832	5.964233	2.07E-30	4.04E-27
ELAVL3	2.614865	-1.16713	3.53E-30	6.20E-27
FCRLB	1.294332	1.213996	9.70E-27	1.55E-23
CLEC4G	2.086696	-1.79792	3.38E-26	4.94E-23
RIMS1	1.631434	-2.311	2.34E-25	3.16E-22
ONECUT2	1.619575	-1.11587	1.04E-24	1.30E-21
AKR1B10	-5.37794	2.210739	1.01E-23	1.18E-20
CACNG7	2.801556	-4.22466	3.96E-23	4.34E-20
VWC2	2.497398	-2.23236	8.12E-23	8.39E-20
IGF2BP1	2.563667	-1.54968	5.58E-22	5.21E-19
NKAIN1	-3.38757	-0.50787	5.64E-22	5.21E-19
PTH2R	1.702502	-2.39657	1.79E-21	1.57E-18
USP41	2.118016	-3.05932	4.45E-21	3.72E-18
MTRNR2L	1.335591	0.10658	1.20E-20	9.61E-18
MYH1	3.697338	-2.30697	5.94E-20	4.53E-17
HOXA10	1.810918	-1.52273	1.88E-19	1.38E-16
ATG9B	1.042006	0.59829	1.40E-18	9.84E-16
DPRX	-8.05081	0.707651	2.13E-18	1.44E-15
IHH	-4.48204	-0.75083	2.42E-18	1.57E-15
AKR7A3	1.105583	-0.36464	5.38E-18	3.37E-15
SFTPA2	2.520364	4.265723	1.06E-17	6.29E-15
ATP2B2	1.499575	-2.18673	1.07E-17	6.29E-15
CYP4F3	-2.7434	-1.20531	2.59E-17	1.47E-14
MNX1	-3.7833	-1.93227	7.77E-17	4.26E-14
PRAP1	-2.50608	-1.51201	1.36E-16	7.24E-14
PITX2	2.124221	-3.30567	2.91E-16	1.50E-13
HOXA9	1.938514	-1.82282	3.16E-16	1.59E-13
ZDHHC22	-2.56557	-1.8389	4.02E-16	1.96E-13
LHX1	-4.89107	-2.14808	4.45E-16	2.11E-13
CAPN8	1.34411	0.759699	4.66E-16	2.15E-13
ODF3L1	-1.48847	-0.16323	6.54E-16	2.87E-13
MYBPH	1.618665	1.515282	9.25E-16	3.96E-13
TNNI2	1.381295	-0.52409	9.78E-16	4.01E-13
PPY	3.311969	2.051738	9.81E-16	4.01E-13
SLC47A2	-1.74234	1.313787	1.03E-15	4.13E-13
TMEM200	1.24327	-0.66146	1.73E-15	6.59E-13
DPYSL5	-3.25183	-2.36401	1.73E-15	6.59E-13
NXF3	1.376026	-2.11594	4.66E-15	1.71E-12
DHRS2	-2.42061	2.107689	4.69E-15	1.71E-12
TNFSF4	1.004522	1.66587	1.34E-14	4.82E-12
AKR1C2	-2.24817	2.788641	1.62E-14	5.68E-12
AKR1C1	-1.72243	2.791105	2.88E-14	9.92E-12
TLX1	-2.67122	-2.37649	3.08E-14	1.04E-11
TNNT3	1.54409	-0.36528	3.89E-14	1.29E-11
IL36G	2.068516	-3.50232	6.73E-14	2.19E-11
FUT9	2.075934	0.019564	6.93E-14	2.21E-11
PNLIP	-6.04436	-2.78604	7.41E-14	2.32E-11
SLC5A5	-2.69457	3.391684	9.59E-14	2.95E-11
AQP8	1.427527	-1.83287	1.22E-13	3.69E-11

CPA1	-3.07688	-2.56846	1.79E-13	5.23E-11
AGRP	1.213955	-2.81382	1.97E-13	5.67E-11
CDHR5	1.202081	-2.27121	2.29E-13	6.48E-11
SEMG1	-5.60323	-2.27527	3.51E-13	9.79E-11
NOTUM	-2.47856	-0.22586	4.08E-13	1.12E-10
VSIG1	1.06504	0.319177	4.20E-13	1.13E-10
NR4A3	-1.53154	4.020959	5.67E-13	1.48E-10
EMX1	1.713288	-3.16587	6.40E-13	1.62E-10
SOX14	-5.5966	-2.35674	6.42E-13	1.62E-10
CALN1	1.922551	-0.55108	6.45E-13	1.62E-10
RBM24	1.13724	-0.4036	6.83E-13	1.69E-10
ADAM12	1.266522	3.203644	7.22E-13	1.76E-10
AMER2	-2.75897	-1.61155	7.47E-13	1.80E-10
HOXA11	1.812535	-1.56918	1.09E-12	2.55E-10
CDKN2A	1.053073	2.426901	1.10E-12	2.55E-10
PTGDS	-1.62674	4.846639	1.19E-12	2.71E-10
TRIM67	1.143271	-2.41801	1.32E-12	2.97E-10
LOXL4	-1.07751	4.589226	1.34E-12	2.99E-10
PTPRN	1.41114	-1.39189	1.37E-12	3.01E-10
ACTN2	1.766648	0.157503	2.76E-12	5.69E-10
SYT1	1.376682	3.22112	4.16E-12	8.40E-10
FGF21	2.669681	-3.93556	4.84E-12	9.64E-10
SEMG2	-5.5249	-2.99369	4.89E-12	9.64E-10
RNF17	-2.3954	-2.11132	4.96E-12	9.68E-10
SLC13A2	2.022884	-3.87062	5.73E-12	1.09E-09
FOXB1	1.881515	-3.60666	5.92E-12	1.12E-09
DKK1	1.270565	0.010135	6.79E-12	1.27E-09
CADPS	-2.07202	0.37913	7.36E-12	1.36E-09
HBEGF	-1.04585	5.850857	8.38E-12	1.52E-09
CACNA1E	1.744856	0.751143	9.63E-12	1.71E-09
MLN	-3.11743	-2.69457	9.80E-12	1.72E-09
EGR2	-1.03854	6.127676	1.24E-11	2.13E-09
KRT23	-2.23305	-1.96478	1.40E-11	2.39E-09
WT1	1.508905	-2.2719	1.47E-11	2.48E-09
SLC13A5	-1.46174	-0.99909	1.85E-11	3.10E-09
PODNL1	1.17893	1.718045	2.47E-11	4.09E-09
PRKCG	-1.90998	-2.60677	2.56E-11	4.19E-09
LMOD3	1.028743	-1.90423	2.73E-11	4.44E-09
SBSPON	-1.05906	1.348102	2.93E-11	4.72E-09
KIRREL3	1.725341	-0.07572	3.30E-11	5.27E-09
POSTN	1.158809	6.850984	3.69E-11	5.83E-09
SFTPA1	2.201958	6.180446	4.25E-11	6.55E-09
COL1A2	1.015064	9.459524	4.36E-11	6.65E-09
PPBP	1.411411	-0.34663	5.07E-11	7.68E-09
ACTA1	1.73666	1.589497	5.51E-11	8.20E-09
DRC1	-1.22072	-2.0295	6.20E-11	9.07E-09
UTS2R	-1.38296	0.537037	6.52E-11	9.47E-09
ATF3	-1.22046	7.154084	7.69E-11	1.10E-08
MYBPHL	1.03085	-0.42493	8.66E-11	1.22E-08
DKK4	-2.3014	-3.5772	9.18E-11	1.28E-08
NEFH	-1.20139	0.840228	9.40E-11	1.29E-08
SCUBE1	-1.32839	3.24135	9.80E-11	1.33E-08
NR0B1	-3.80064	-2.01217	9.82E-11	1.33E-08
TMEM132	-2.15042	-0.36671	1.21E-10	1.62E-08
AZU1	1.268487	-1.0044	1.28E-10	1.69E-08
AGR3	-1.48502	-0.75807	1.46E-10	1.88E-08
COL9A3	-2.17626	6.866388	1.63E-10	2.08E-08
TDRD1	1.143135	-3.06275	2.64E-10	3.34E-08

FAT3	1.102313	-2.08369	2.72E-10	3.41E-08
EPHA5	-1.82029	-0.00214	2.79E-10	3.47E-08
KRT85	-2.2794	-1.47119	2.97E-10	3.66E-08
ARSF	2.193661	-0.0625	2.98E-10	3.66E-08
GUCA1A	1.47873	-4.0714	3.14E-10	3.80E-08
KCNJ6	1.302079	-2.13998	3.24E-10	3.89E-08
CCN4	1.136583	1.165763	3.51E-10	4.16E-08
POMC	-1.04322	0.534223	3.93E-10	4.63E-08
CTHRC1	1.074308	3.959736	4.14E-10	4.84E-08
COL3A1	1.028351	9.283793	4.17E-10	4.85E-08
MCEMP1	1.349182	1.285154	4.73E-10	5.39E-08
SMCO3	-1.1167	-0.75584	6.32E-10	7.07E-08
GLRA1	-2.25141	-2.96996	6.59E-10	7.28E-08
GPX2	-1.13588	-1.44182	9.22E-10	9.95E-08
HSD17B2	1.507108	-3.53567	9.23E-10	9.95E-08
MT3	1.29113	0.002911	9.71E-10	1.04E-07
COL11A1	1.675886	3.906935	1.01E-09	1.07E-07
ATP2B3	2.483417	-0.55648	1.10E-09	1.15E-07
COL1A1	1.103214	10.02343	1.18E-09	1.22E-07
CPB1	-2.47629	-3.25602	1.22E-09	1.25E-07
WIF1	-3.44233	1.868944	1.25E-09	1.28E-07
CRCT1	1.806189	-4.2614	1.29E-09	1.31E-07
FREM3	1.304557	-1.54781	1.35E-09	1.36E-07
DPYS	-1.50097	-2.26082	1.56E-09	1.56E-07
HMGCS2	-2.53755	-0.16047	1.68E-09	1.66E-07
GAPDHS	-2.06432	-3.53128	1.73E-09	1.69E-07
STAB2	1.152542	-0.81074	1.74E-09	1.69E-07
FOSB	-1.2679	8.235626	2.23E-09	2.16E-07
MYL1	3.243552	-0.47217	2.38E-09	2.28E-07
SLC35F1	-1.07222	1.259841	2.45E-09	2.34E-07
SLC24A2	1.568368	-1.11926	2.86E-09	2.67E-07
CELA3B	-3.33125	-3.64991	3.29E-09	3.05E-07
CELA3A	-3.66933	-3.54712	3.31E-09	3.06E-07
CYP26A1	1.170495	-1.11009	3.50E-09	3.21E-07
PRAMEF1[illegible]	1.755269	-3.69746	3.55E-09	3.22E-07
SLC35D3	1.348503	-3.12123	3.56E-09	3.22E-07
SERPINB1[illegible]	1.513894	-4.07521	3.57E-09	3.22E-07
IGFL3	1.649439	-3.77967	3.66E-09	3.28E-07
IL37	1.772554	-1.99351	4.15E-09	3.66E-07
EPPIN	-1.7327	-2.32343	6.06E-09	5.17E-07
AKR1C3	-1.0239	3.203833	7.00E-09	5.71E-07
NR0B2	1.347463	-1.38793	7.17E-09	5.83E-07
LHFPL4	1.179668	-1.07231	7.30E-09	5.91E-07
CCDC198	-2.20259	-3.44514	7.75E-09	6.21E-07
RETN	1.317759	-0.26797	7.91E-09	6.29E-07
CTRB2	-1.56175	-3.21406	8.02E-09	6.34E-07
IVL	1.431597	2.818414	9.89E-09	7.76E-07
TAGLN3	-1.48461	0.860495	1.01E-08	7.86E-07
PSG6	1.629045	-3.09172	1.01E-08	7.86E-07
PSG1	1.330233	1.585243	1.05E-08	8.09E-07
LRRC15	1.457607	3.206677	1.16E-08	8.89E-07
CHRND	1.507837	-3.56431	1.30E-08	9.91E-07
USH1C	-1.38686	-0.49046	1.36E-08	1.03E-06
TGM4	-1.41593	-2.15004	1.36E-08	1.03E-06
CNTNAP2	-1.43145	1.506217	1.84E-08	1.34E-06
LCN1	1.151471	-4.30144	1.97E-08	1.43E-06
IL12RB2	-1.10394	-0.9063	2.15E-08	1.54E-06
ALDH1L1	1.114461	2.532548	2.24E-08	1.59E-06

VAX1	-3.30128	-3.36455	2.47E-08	1.74E-06
HOXB8	1.224465	-3.24556	2.62E-08	1.83E-06
CXCL5	1.236703	0.135281	3.33E-08	2.22E-06
CASR	-2.48929	-2.17039	3.60E-08	2.34E-06
CBLN2	1.278798	-3.62489	3.87E-08	2.50E-06
GPR142	-1.45242	-3.04139	4.70E-08	2.96E-06
TNP1	-1.44715	-4.06114	5.27E-08	3.27E-06
CTRB1	-1.52681	-3.70715	5.53E-08	3.39E-06
ATP1B4	1.413653	-3.82912	6.38E-08	3.82E-06
PRRT4	-1.57609	1.813975	8.21E-08	4.76E-06
HYAL4	-1.7031	-3.44036	8.81E-08	5.04E-06
WFDC6	-1.96449	-3.75653	8.84E-08	5.04E-06
PLPPR5	1.573889	-3.88686	9.44E-08	5.31E-06
FER1L6	-1.64581	0.357169	1.01E-07	5.66E-06
CABP7	-1.1173	-0.22645	1.03E-07	5.76E-06
INSL6	-1.90513	-3.75506	1.04E-07	5.76E-06
IGF1	-1.02111	1.188494	1.12E-07	6.22E-06
THEG	1.380432	-2.37451	1.14E-07	6.28E-06
NYAP2	-1.6916	-0.56612	1.18E-07	6.48E-06
MYPN	1.628061	-3.13441	1.21E-07	6.56E-06
PLA2G2A	1.340038	2.756106	1.61E-07	8.48E-06
ATP6V0A4	-1.45388	-2.04972	1.69E-07	8.87E-06
EIF4E1B	1.66809	-3.55742	1.80E-07	9.33E-06
GMNC	-1.73045	-2.14821	1.87E-07	9.63E-06
MYBPC1	1.368607	0.753362	2.03E-07	1.03E-05
NRAP	2.048101	0.081126	2.11E-07	1.07E-05
SOX11	1.023772	0.935903	2.55E-07	1.26E-05
HS3ST4	-1.34415	-2.20616	2.65E-07	1.30E-05
OTOG	-1.59719	-4.22069	3.58E-07	1.72E-05
TFF3	-1.41448	6.560517	3.78E-07	1.81E-05
IGFN1	1.262344	6.655411	3.88E-07	1.85E-05
SLC6A2	-1.7707	-3.07277	4.14E-07	1.95E-05
BMP8A	-1.28817	2.892017	4.16E-07	1.96E-05
SLN	1.778909	-1.17663	4.32E-07	2.01E-05
EPYC	1.714039	-0.00121	4.49E-07	2.08E-05
F5	1.124429	4.283755	4.92E-07	2.25E-05
SCRT2	2.115075	-3.13586	5.26E-07	2.37E-05
NUGGC	-1.00751	1.214391	5.63E-07	2.52E-05
ZIC4	1.438596	-4.29365	5.67E-07	2.53E-05
MPPED1	-2.64809	-2.177	5.69E-07	2.53E-05
OLFM4	1.170647	-0.8489	5.76E-07	2.55E-05
UGT3A1	-2.56161	-3.24391	5.85E-07	2.57E-05
KRT75	-2.1453	-2.59324	6.61E-07	2.86E-05
MUC21	1.147703	4.373301	6.94E-07	2.98E-05
CACNA1S	1.434106	-2.73144	7.24E-07	3.06E-05
INSM2	1.01879	-4.08076	7.35E-07	3.08E-05
OR8G5	1.390924	-4.29834	7.37E-07	3.08E-05
CELA2A	-1.37151	-3.72885	8.26E-07	3.40E-05
PVALB	1.459885	1.855102	8.63E-07	3.53E-05
CLCA2	-1.62333	1.144207	8.67E-07	3.54E-05
HECW1	-1.12134	-0.28364	9.00E-07	3.61E-05
COL10A1	1.084933	3.573767	9.40E-07	3.73E-05
NDST4	-2.04851	-3.9342	9.99E-07	3.91E-05
MMP13	1.699828	1.500363	1.00E-06	3.92E-05
C6orf118	-1.01059	-3.32771	1.03E-06	4.00E-05
CPLX2	-1.65943	-1.00924	1.09E-06	4.21E-05
TRH	-1.0897	-3.22489	1.24E-06	4.66E-05
ELANE	1.151585	-1.60612	1.25E-06	4.69E-05

MFAP5	1.025516	2.572742	1.28E-06	4.81E-05
REG1A	-3.16317	-1.58886	1.53E-06	5.63E-05
DPPA5	-1.77345	-4.11463	1.72E-06	6.19E-05
PPP1R3A	2.427913	-4.224	1.77E-06	6.35E-05
NPY	-2.10438	2.286182	1.77E-06	6.36E-05
SPATA19	-1.37083	-3.58005	1.80E-06	6.45E-05
CRH	1.077602	-3.3345	1.82E-06	6.49E-05
SPRR1B	1.479587	-0.4968	1.83E-06	6.49E-05
SLC5A7	-1.76577	-0.53316	1.84E-06	6.54E-05
TFF2	-1.75417	-2.08087	2.05E-06	7.14E-05
C10orf71	2.101628	-3.15798	2.07E-06	7.21E-05
SERPINB7	1.110466	-3.0688	2.14E-06	7.40E-05
TTC29	-1.00013	-3.17414	2.25E-06	7.68E-05
SMPX	1.512387	-2.98343	2.36E-06	8.03E-05
PTPRT	-1.30391	-0.29063	2.59E-06	8.69E-05
LGR5	1.173411	-0.58955	2.64E-06	8.82E-05
ZIC1	1.341877	-3.62615	2.89E-06	9.44E-05
ADIPOQ	1.817601	-1.70031	3.04E-06	9.78E-05
IL36A	1.166569	-3.95372	3.39E-06	0.000106174
METTL11E	1.528161	-4.15015	3.45E-06	0.00010765
AADAC	-1.4458	-3.87148	3.55E-06	0.000110204
HOXA6	1.056082	-4.39381	3.63E-06	0.000112054
CIDEC	1.214841	-2.81086	3.68E-06	0.000113264
LIPK	1.426895	-3.94407	4.02E-06	0.000122693
CLPS	-2.44583	-4.34475	4.35E-06	0.000131695
CSMD3	-1.17014	-3.04752	4.39E-06	0.00013255
MUCL1	-2.08674	-3.75548	4.59E-06	0.000136932
KCNJ13	-1.0162	1.056819	5.04E-06	0.00014807
ZNF280A	-1.52363	-4.0761	5.43E-06	0.000157274
ADARB2	1.194959	-0.53542	5.51E-06	0.000158893
NOX5	-1.01895	2.429598	5.53E-06	0.000159171
KLK1	1.31079	3.54145	5.86E-06	0.000167424
SCN1A	1.07816	-2.97332	6.07E-06	0.000171647
TRARG1	1.039268	-2.83236	6.40E-06	0.000178666
PLA2G2D	-1.17052	1.13358	6.49E-06	0.000180999
SERPINB1	2.346312	0.064087	8.93E-06	0.000234661
PGLYRP4	1.238806	-3.99976	8.96E-06	0.000235249
CIDEA	1.129563	-3.73133	9.12E-06	0.00023853
BIRC7	1.00587	3.116145	9.60E-06	0.000247062
ABCC12	-1.60317	-1.52611	9.74E-06	0.000250141
MYF6	1.756603	-3.50877	1.05E-05	0.00026452
TYR	-1.48214	-4.01216	1.11E-05	0.000277892
ARMC3	-1.24817	-2.51855	1.26E-05	0.000307508
KERA	1.296368	-3.14672	1.28E-05	0.000311449
KRT5	-1.53988	3.257212	1.30E-05	0.000313298
CSRP3	2.078827	-1.89095	1.38E-05	0.000330064
CT83	2.173071	-4.22254	1.38E-05	0.000330486
ANGPTL7	-1.10883	-2.88939	1.46E-05	0.000345184
TMPRSS1	1.103464	1.791694	1.47E-05	0.000345419
HOXD10	1.103116	-1.56324	1.50E-05	0.000350972
MUC5AC	-1.16268	-3.82944	1.53E-05	0.000356566
SPATA22	-1.28154	-3.49605	1.54E-05	0.000357259
GDF10	-1.09119	1.229175	1.77E-05	0.000398467
CD5L	1.201619	-1.59713	1.84E-05	0.000411309
KCNC2	-1.95281	-4.27469	2.11E-05	0.000460125
IGLL5	-1.40946	5.893142	2.13E-05	0.000463154
PROKR1	-1.04666	-3.91095	2.32E-05	0.000498184
SERPIND1	-1.12674	-1.00923	2.64E-05	0.000555116

SERPINA9	-1.7924	-1.69197	3.30E-05	0.00066018
VIL1	-1.022	-3.39649	3.72E-05	0.000726504
FGFBP1	-1.06326	3.121615	3.93E-05	0.00075581
SPRR2D	1.307323	-2.74432	4.17E-05	0.000787344
KLK15	1.165313	-0.61413	4.21E-05	0.000792062
SPRR3	1.280391	-2.96367	4.30E-05	0.00080629
WFDC8	-1.09792	-2.92294	4.49E-05	0.000837263
SBSN	-1.19616	0.620491	4.58E-05	0.00084839
MSLN	-1.02356	1.700592	4.93E-05	0.000899718
GPR15	1.000219	-3.34216	5.52E-05	0.000988919
MYMK	-1.03877	-3.72493	5.57E-05	0.000996393
SKOR2	-1.8109	-1.71782	5.58E-05	0.000997407
KCNJ3	-1.29598	-1.19376	5.84E-05	0.001033413
APOA1	-1.08408	2.228829	5.91E-05	0.0010429
LGALS14	-1.20625	-4.36193	6.04E-05	0.001062295
COL6A5	1.383971	0.770182	6.07E-05	0.001066166
BRS3	-1.51507	-3.61631	6.40E-05	0.00111217
PTH	-2.8531	-2.22005	6.87E-05	0.001174829
PLD5	-1.01311	-1.96505	6.89E-05	0.001175874
NEUROG2	1.245874	-2.50549	6.99E-05	0.001189676
NTRK1	1.151062	0.510774	7.59E-05	0.001270438
MYOG	1.279982	-4.12657	7.73E-05	0.00128904
SLC14A2	-1.10329	2.477558	7.84E-05	0.001299993
TMEM213	-1.53881	-1.8784	8.01E-05	0.001319218
SALL3	-1.35066	-1.40184	8.08E-05	0.001327574
HP	1.094275	2.392228	8.08E-05	0.001327574
SLC18A3	1.257073	0.780866	8.36E-05	0.001356461
WNT3A	-1.02408	-2.27225	8.41E-05	0.001361594
SPOCK3	-1.12325	-0.20108	9.14E-05	0.001453714
PSG8	1.22727	1.801672	9.28E-05	0.001471232
RTP5	-1.02447	-3.20295	9.72E-05	0.001520986
IGLL1	-1.04112	-2.34772	9.78E-05	0.00152682
APELA	1.23213	-1.99366	0.000106975	0.001639076
KRT17	-1.07193	5.293989	0.000110285	0.001679531
GP2	-1.92433	-3.68379	0.000129291	0.001891855
SRARP	-1.06626	-1.90584	0.000135466	0.001957429
MYOC	-1.15649	0.70954	0.000143196	0.002042556
GIP	-1.43558	-4.08198	0.000146115	0.002073965
TCL1A	-1.27354	0.228913	0.000157834	0.002204786
ASB5	1.366261	-3.10246	0.00016169	0.002246134
PPP2R2C	-1.11008	0.655994	0.000168247	0.002309815
TFF1	-1.1747	-2.60476	0.000185707	0.002506409
MYH6	1.154665	-2.38191	0.000187879	0.002533769
RGS13	-1.03899	-1.25449	0.000194593	0.002612277
CTXN3	1.080269	-4.25917	0.000197532	0.002641635
TERB2	-1.15193	-4.05392	0.000198145	0.002647809
CHRNB3	-1.51403	-4.45019	0.000261621	0.003321622
KRT84	-1.08235	-4.35379	0.000292915	0.003630129
FCAMR	-1.2334	-1.02124	0.000346153	0.004115165
SMR3B	-1.66676	1.101343	0.000358495	0.004238929
GPRC6A	1.072812	-4.30188	0.00036094	0.004262099
SLC5A1	-1.02172	-0.61595	0.000376153	0.00440325
IRX6	-1.14183	1.309712	0.000392202	0.004542711
GCGR	1.097289	0.806319	0.000409844	0.004712807
LDLRAD1	-1.0392	-3.71709	0.000441179	0.004984984
CR2	-1.00803	2.737921	0.000470749	0.005241525
XIRP2	1.690217	-0.09596	0.000472344	0.005255949
C14orf180	1.021767	-2.87484	0.000492891	0.00542953

R3HDML	-1.1227	-3.70641	0.000497501	0.005466591
PAX7	1.474889	-3.25507	0.000507077	0.00554061
NLRP4	-1.02061	-3.32697	0.000540698	0.005816916
AICDA	-1.15004	-2.25029	0.000579364	0.006120974
SMYD1	1.583749	-3.05381	0.000590503	0.006205053
CD8B2	-1.14267	-2.7317	0.000607901	0.006338564
VGLL2	1.429057	-4.3826	0.000615975	0.006403736
TBR1	1.015921	-3.90244	0.00065401	0.006688271
NWD2	1.244895	-0.48911	0.000711085	0.00715117
PNLDC1	-1.01165	-1.11528	0.000721442	0.007230482
CALML3	-1.28747	-0.97068	0.00079501	0.007855704
MZB1	-1.01251	4.286035	0.000855284	0.008297203
CHGA	-1.38994	-1.43628	0.000882631	0.008506099
FGG	-1.61518	-2.90841	0.001039289	0.00955439
OBP2B	1.03037	-0.44237	0.001108136	0.010014289
SORCS3	1.017653	-4.03005	0.001130784	0.010135496
SP8	-1.29614	-3.47673	0.00118599	0.010485803
KLHL40	-1.06923	-3.13231	0.001393077	0.011891609
FCRL4	-1.04912	-3.06939	0.001441513	0.01223994
CBY2	-1.00192	-3.22371	0.001487997	0.012567458
FGB	-1.71077	-2.61904	0.001505656	0.012692178
CHAT	1.05499	-2.40146	0.001597744	0.013260611
HOXC10	-1.15624	-1.53107	0.00183385	0.014565289
FGA	-1.977	-2.21907	0.001886844	0.01487701
SCGB3A2	-1.07102	-1.71658	0.002589461	0.018921494
MTRNR2L	-1.11003	4.232446	0.002784739	0.020015238
SST	1.063154	-0.64483	0.003590828	0.024391235
AGXT	-1.04125	-3.85258	0.004472171	0.028482717
CARTPT	1.106871	4.031383	0.005238059	0.031869394
KRT6A	-1.01989	3.195192	0.005400123	0.032562076
ZFP42	-1.05885	-3.14412	0.006941774	0.039029972
APOH	-1.17028	-3.20518	0.007362097	0.040689662
ALPI	1.112214	-4.20212	0.008165935	0.043902527
TTR	-1.15362	-1.38436	0.008406334	0.044878936
