# Supplementary material for: Construction and Evaluation of a Tumor Mutation Burden-Related Prognostic Signature for Thyroid Carcinoma
Source: Comput Math Methods Med. 2021 Oct 14;2021:1435827. doi: 10.1155/2021/1435827 (PMC8538398; doi:10.1155/2021/1435827)
Supplement: Supplementary 2 — Supplementary Table 2: univariate Cox regression analysis on TMB-related DEGs. [file 1435827.f2.pdf]

gene	HR	HR.95L	HR.95H	pvalue
COL10A1	1.018946	1.005684	1.032383	0.004986
BMP8A	1.006256	1.000252	1.012296	0.04111
MFAP5	1.030532	1.004945	1.05677	0.019054
PODNL1	1.051077	1.016218	1.087132	0.003793
WNT3A	1.126717	1.037947	1.22308	0.004379
F5	1.01179	1.004456	1.019178	0.00159
EPPIN	1.367848	1.050299	1.781404	0.020121
SERPIND1	1.082392	1.027453	1.140269	0.002892
C6orf118	6.806156	1.6444	28.17061	0.008139
ADARB2	1.320935	1.085581	1.607315	0.005433
AGRP	1.176733	1.088437	1.272192	4.33E-05
MCEMP1	1.034247	1.01826	1.050485	2.27E-05
SALL3	1.062311	1.023206	1.102911	0.001584
RETN	1.041271	1.017293	1.065815	0.000668
SRARP	1.79581	1.306105	2.469122	0.000314
EPYC	1.035503	1.004762	1.067185	0.023274
CAPN8	1.105613	1.046574	1.167983	0.000336
PTGDS	1.000962	1.000311	1.001614	0.003779
WT1	2.196292	1.078232	4.473713	0.030199
DRC1	1.995731	1.070498	3.720647	0.029681
TMPRSS1	1.023281	1.004385	1.042532	0.015515
PROKR1	1.769875	1.259488	2.487086	0.001005
HMGCS2	1.013357	1.001845	1.025	0.022833
TFF3	1.000765	1.000291	1.001239	0.001571
PPBP	1.027873	1.015098	1.04081	1.65E-05
CXCL5	1.170985	1.097672	1.249195	1.71E-06
STAB2	2.758467	1.124647	6.765803	0.026652
ARMC3	1.693429	1.189631	2.410581	0.003458
MYH6	2.207772	1.077167	4.525073	0.030543
TFF1	1.214818	1.106725	1.333468	4.26E-05
SCN1A	42.24761	1.236509	1443.468	0.037728
FUT9	1.320715	1.063756	1.639746	0.011741
OLFM4	1.033106	1.00991	1.056834	0.004938
